# Molecular Biomarkers for Timely and Personalized Prediction of Maternal-Fetal Health Risk

**DOI:** 10.3390/biom15030312

**Published:** 2025-02-20

**Authors:** Daniel Estrela, Rita F. Santos, Alice Masserdotti, Antonietta Silini, Ornella Parolini, Inês Mendes Pinto, Andrea Cruz

**Affiliations:** 1 International Iberian Nanotechnology Laboratory (INL), 4715-330 Braga, Portugal; daniel.estrela.76@gmail.com; 2 Institute for Research and Innovation in Health (i3S), University of Porto, 4200-135 Porto, Portugal; ritafsantos01@hotmail.com (R.F.S.); ines.pinto@i3s.up.pt (I.M.P.); 3 Molecular and Analytical Medicine Laboratory, Department of Biomedicine, Faculty of Medicine, University of Porto, 4200-319 Porto, Portugal; 4 Department of Life Science and Public Health, Università Cattolica del Sacro Cuore, 00168 Rome, Italy; alice.masserdotti@unicatt.it (A.M.);; 5 Centro di Ricerca E. Menni, Fondazione Poliambulanza Istituto Ospedaliero, 25124 Brescia, Italy; antonietta.silini@poliambulanza.it; 6 Fondazione Policlinico Universitario “Agostino Gemelli” IRCCS, 00136 Rome, Italy

**Keywords:** biomarker profiling, placental dysfunctions, early diagnosis, disease management

## Abstract

Molecular biomarker profiling is an emerging field in maternal-fetal health with the potential to transform early detection and prediction of placental dysfunction. By analysing a range of biomarkers in maternal blood, researchers and clinicians can gain crucial insights into placental health, enabling timely interventions to enhance fetal and maternal outcomes. Placental structural function is vital for fetal growth and development, and disruptions can lead to serious pregnancy complications like preeclampsia. While conventional methods such as ultrasound and Doppler velocimetry offer valuable information on fetal growth and blood flow, they have limitations in predicting placental dysfunction before clinical signs manifest. In contrast, molecular biomarker profiling can provide a more comprehensive assessment by measuring proteins, metabolites, and microRNAs (miRNAs) in maternal blood, reflecting the placenta’s endocrine and metabolic functions. This approach offers a deeper understanding of placental health and function, aiding in early detection and prediction of complications. Challenges in developing molecular biomarker profiling include pinpointing specific molecular changes in the placenta linked to pathologies, timing predictions of conditions before clinical onset, and understanding how placental dysfunction affects maternal metabolism. Validating specific biomarkers and integrating them effectively into clinical practice requires further research. This review underscores the significance of molecular biomarker profiling as a powerful tool for early detection and prediction of placental dysfunction in maternal-fetal health. Through an exploration of biomarker analysis, we delve into how a deeper understanding of placental health can potentially improve outcomes for both mother and baby. Furthermore, we address the critical need to validate the utility of biomarkers and effectively integrate them into clinical practice.

## 1. Introduction

Molecular biomarker profiling is rapidly emerging as a promising field in maternal-fetal health, with the potential to revolutionize early detection and prediction of placental dysfunction. This innovative approach involves identifying specific biological markers at the molecular level, such as proteins, RNA, or metabolites, which provide insights into the health and function of the placenta. Given the critical role the placenta plays in supporting fetal development and ensuring maternal well-being, early identification of dysfunctions through biomarker profiling could significantly improve prenatal care. By enabling timely interventions, this approach holds the promise of reducing pregnancy complications and improving outcomes for both mother and baby.

## 2. Placenta

### 2.1. Structure and Cellular Components of Human Term Placenta

The placenta is a unique fetal-maternal organ composed by complementary but distinct fetal and maternal tissues, such as the fetal membranes, the chorionic villi, the umbilical cord, and the decidua [1,2].

The fetal membranes include the amniotic and the chorionic membranes, namely the amnion and the chorion, respectively [1,3]. They protect and support the growth of the foetus by serving as a semi-permeable physical barrier that withstands mechanical stress from fetal movements, regulates amniotic fluid volume, and shields against local insults (i.e., infection) [4,5]. The amnion, the innermost membrane, is a thin, avascular structure composed of an epithelial monolayer, in direct contact with the amniotic fluid, and a mesenchymal layer, characterized by collagens, rare macrophages and dispersed fibroblast-like mesenchymal cells [1,6]. On the other hand, the chorion is made up of a reticular portion, containing chorionic mesenchymal stromal cells, and a chorionic trophoblastic region, rich in proliferating extravillous trophoblasts.

Chorionic villi, the placenta’s functional units, anchor it to the maternal endometrium and facilitate fetal-maternal exchange [2,3,7]. These finger-like projections contain two layers of specialized epithelial cells: the outer syncytiotrophoblast, which interacts with maternal blood, and the inner cytotrophoblast, which gradually shrinks throughout pregnancy [8].

Developing from the chorionic plate, the umbilical cord connects the fetus to the placenta, facilitating Fetal-maternal exchanges without direct blood mixing [9]. It is made up of a sheath of amniotic epithelial cells, an internal fibroblast-like cells enriched connective tissue (Wharton’s jelly) [10], one vein (conveying oxygenated and nutrient-rich blood) and two arteries (transporting de-oxygenated and nutrient-depleted blood) [11,12].

The maternal component of placenta, the decidua, is crucial for embryo implantation and pregnancy maintenance until placenta formation [13]. It consists of three different regions, rich in proliferating functional endometrial cells, named decidua basalis, decidua capsularis and decidua parietalis [14,15,16].

### 2.2. Function of Human Placenta

The placenta is the very first organ to be formed during pregnancy and although it is temporary, it plays a fundamental role as a lifeline between the mother’s bloodstream and the foetus. Placenta performs various functions aimed at ensuring the successful growth of the embryo and the foetus and in order to fulfil its functions, the placenta undergoes structural adaptations during pregnancy. As a matter of fact, alterations in the development of the placental structure may seriously compromise outcomes of the pregnancy [17].

(i) The placenta provides the foetus with oxygen and nutrients, acting like a lung in the exchange of oxygen and carbon dioxide and working as a digestive system absorbing all necessary nutrients for the growth and development of the foetus. The placenta has two separate circulatory systems, as it receives blood supply from both the mother and the foetus. Deoxygenated blood flows through the umbilical arteries from the foetus to the placenta, where it is oxygenated by the maternal blood flow, and then returns through the umbilical veins to the foetus. The transfer of nutrients occurs in the same way that oxygen and carbon dioxide are transferred in the placenta [17]. (ii) Placenta removes also harmful waste, functioning as a kidney by transferring all Fetal waste, via the umbilical cord, to the mother’s veins [18]. (iii) It behaves as an immune filter that leads the mother’s antibodies to the foetus thus providing immune protection within the first months of life, but also producing anti-inflammatory hormones and cytokines that sustain immunological tolerance and buffer maternal immune cell invasion [19]. (iv) Moreover, it acts as a semi-permeable physical barrier, preventing potentially toxic substances and pathogens from reaching the foetus [20,21]. (iv) Finally, the placenta operates as an endocrine organ, producing and secreting many hormones, growth factors, cytokines and chemokines that regulate the course of pregnancy, support the foetus growth, promote maternal adaptation to pregnancy and induce labour onset at the end of gestation [22,23].

## 3. Prenatal Screening for Placenta Dysfunction

Prenatal screening for placental dysfunction involves various tests and assessments to detect issues with the placenta during pregnancy. This screening involves a combination of ultrasound monitoring, advanced imaging techniques, biochemical tests, and consideration of clinical and methodological factors to detect and manage conditions like placental insufficiency, ensuring the well-being of both the mother and the foetus throughout pregnancy [24,25,26]. Ultrasound monitoring is a primary tool for assessing placental health. It can measure the size, shape, and echotexture of the placenta, providing important information about its morphology. While most studies use 2D ultrasound to evaluate placental morphology, newer studies have utilized 3D techniques, which can offer more detailed information about the placenta’s structure and function [24,27,28].

Biochemical tests potentially offer a more comprehensive and objective assessment of placental health by measuring various placental factors in maternal biofluids. These tests reflect the endocrine and metabolic functions of the placenta, enabling earlier detection of placental dysfunction, particularly in low-resource settings with limited access to high-quality ultrasound equipment and trained operators [24]. Biochemical tests can be performed at any stage of pregnancy, unlike ultrasound assessment of fetal size, typically performed in the second and third trimesters.

The most commonly used biochemical tests in prenatal screening for placental dysfunction include those that measure placental products, such as proteins, peptides, metabolites, and hormones, in maternal biofluids, like serum, plasma, or urine [24,29,30]. This can allow earlier detection of placental dysfunction, which may enable more timely interventions to improve Fetal and maternal outcomes.

These tests are based on the hypothesis that levels of such products in maternal fluids reflect endocrine and metabolic functions of the placenta. Many placental products can be detected in maternal biofluids, including protein hormones such as human chorionic gonadotropin (hCG), human placental lactogen (hPL), human placental growth hormone (hPGH), placental growth factor (PlGF), placental protein-13 (PP-13), soluble fms-like tyrosine kinase-1 (sFlt-1), pregnancy-specific glycoproteins, and steroid hormones including estrogens and progesterone with their related metabolites [29].

However, it is important to note that these biochemical tests are primarily used in prenatal screening to indicate the risk of conditions like Down syndrome and spina bifida, rather than directly assessing placental dysfunction [24,30]. Therefore, clinical and methodological factors must also be considered when assessing the risk of placental dysfunction. Furthermore, in healthy women, bioactive peptides, such as cytokines, play an important role in inducing physiological adaptations required for a successful pregnancy. However, when inappropriately regulated, they lead to disease [31]. Thus, their detectability at earlier stages of gestation gives them predictive value, establishing them as biomarkers for pregnancy complications [32].

Current biochemical tests for placental function have limitations in diagnosing placental dysfunction. Firstly, the evidence supporting their effectiveness is generally of low or very low quality, with insufficient evidence to draw conclusions about their impact on reducing the number of small for gestational age (SGA) babies or neonatal intensive care unit admissions [24,30,33]. Secondly, the interpretation of biochemical test results can be challenging due to the lack of standardization in measuring placental factors. Different experimental approaches, such as enzyme-linked immunosorbent assay (ELISA), mass spectrometry, or point-of-care tests, can yield varying results, making comparing and interpreting data across studies difficult. Lastly, the timing of biochemical tests is crucial, as placental factors change throughout pregnancy. For example, some studies have found that elevated sFlt-1 in the first trimester is associated with a reduced risk of SGA infants and stillbirth associated with a placental cause, while high PlGF in the first trimester is associated with a reduction in SGA infants. However, the predictive ability of these biomarkers in late pregnancy is still being evaluated [24]. These results highlight the crucial necessity for validating molecular biomarkers that can accurately assess various stages of placental development and function.

## 4. Molecular Signatures of Placenta-Related Diseases

Despite advancements in modern healthcare, the global maternal and fetal mortality rate remains unacceptably high, with an estimated 287,000 women dying during and following pregnancy and childbirth in 2020. Such consequences happen due to several factors that are known to increase the risk of developing a pregnancy complication. These include exposure to environmental pollutants and maternal health factors, such as poor nutrient intake, poor cardiovascular or metabolic health, family/previous pregnancy history, asthma, maternal obesity, and advanced maternal age [32,34,35,36]. The primary complications contributing to 75–80% of all maternal deaths are preeclampsia (PE), severe bleeding, and infections, which often arise during pregnancy, childbirth, and the postpartum period. However, it is known that maternal health factors, such as advanced maternal age, are associated not only with the development of PE but also with other adverse pregnancy outcomes, like miscarriage (spontaneous loss of a pregnancy before the 20th week), stillbirth (birth of a baby without any signs of life after 20 weeks of gestation), preterm labour (or pre-term birth, birth of a baby before completing 37 weeks of gestation), intrauterine growth restriction (IUGR), and gestational diabetes mellitus (GDM) [37,38].

All these factors impact the development trajectory of the foetus, potentially resulting in fetal alterations and eventually loss [32,34]. However, many of these deaths could be prevented or treated if diagnosed earlier, highlighting the importance of timely and accurate diagnosis and intervention.

### 4.1. Asthma

Asthma is an inflammatory disorder of the airways that physiologically results in hyperreactivity and clinically, in recurrent episodes of wheezing, chest tightness, or coughing [39]. This disorder is one of the most common chronic disorders that complicate pregnancies, and its prevalence during pregnancy ranges from 3 to 6%. Among those pregnancies, 19% had severe asthma and 16% had poorly controlled asthma [40].

During pregnancy, oxygen and metabolic rate consumption increase by 20%, and the respiratory system undergoes adaptations with significant anatomic and hormonal changes that affect pulmonary function parameters in the mother [40]. Thus, the presence of asthma during pregnancy can result in increased maternal systemic inflammation (inflammation throughout the entire maternal body) and oxidative stress, as well as reduced levels of maternal oxygen especially when asthma is recurrently uncontrolled or when women experience an acute exacerbation of asthma. It is known that this disorder can alter the expression of baseline placental cytokine messenger ribonucleic acid (mRNA), including interleukin (IL)-6 and chemokine IL-8 and can potentially contribute to preterm birth and growth restriction [35].

### 4.2. Obesity

Obesity is a disease defined by an excess accumulation of fat and a body mass index (BMI) over 30 kg/m^2^ [41,42]. In developed nations, one-third of pregnant women are overweight or obese. Maternal obesity is associated with exaggerated physiological changes, including alterations in lipoprotein (complex molecule composed of lipids (fats) and proteins) levels and enhanced gluconeogenesis (process of generating glucose), which place the mother and the child at risk, with the most common adverse outcome being macrosomia (high birth weight) [36].

Maternal obesity also associated with chronic low-grade inflammatory state, which is a consequence of the increased levels of fetal circulating inflammatory markers, altering the pro- and anti-inflammatory balance and decreasing the immune response to pathogens [41]. Furthermore, this disease has been characterised by mitochondrial dysfunction, leading to an increased production of reactive oxygen species (ROS), and altering the secretion of some cytokines, such as IL-6 and IL-8 [41,43].

### 4.3. Preterm Labour (PTL)

Spontaneous preterm labour with intact membranes (PTL), before the 37th week of pregnancy when the amniotic membranes surrounding the baby have not ruptured) represents a clinical phenotype of spontaneous preterm birth and is responsible for approximately one-third of all preterm deliveries. PTL has been linked to intra-amniotic inflammation, which is characterised by elevated amniotic fluid concentrations of inflammatory mediators, such as cytokines and chemokines, and an increased number of immune cells in the amniotic fluid [44]. Furthermore, intra-amniotic infection in pregnancies with PTL is associated with a stronger intra-amniotic inflammatory response compared to that observed during sterile intra-amniotic inflammation. This inflammation is characterised by an increased concentration of IL-6, IL-8 and CCL-3. These lead to the recruitment of neutrophils and monocytes/macrophages [44,45].

### 4.4. Preeclampsia (PE)

Preeclampsia (PE) is a severe complication of pregnancy, leading to maternal and perinatal morbidity and mortality worldwide. It causes over 70,000 maternal and 500,000 infant deaths annually, many of which could be prevented with early diagnosis. PE survivors (over 300 million women and children globally) have reduced life expectancy, and face increased risks of stroke, cardiovascular disease, and diabetes later in life [46]. Babies born from PE-complicated pregnancies may develop neurodevelopmental delays and cardiovascular/metabolic diseases in adulthood [47].

At the pathophysiological level, preeclampsia is characterised by a placental disease involving an imbalance of angiogenic factors that lead to vascular inflammation, endothelial dysfunction, and maternal vascular injury [48]. This imbalance includes a shift in the T cell profile towards a predominance of T helper 1 cells and their associated cytokines, likely contributing to poor placentation, maternal inflammation, and endothelial dysfunction. Placenta-derived factors, such as pro-inflammatory cytokines and anti-angiogenic molecules, can impact the maternal vascular endothelium, triggering the release of additional factors that exacerbate dysfunction. Concurrently, there is a suppression of pro-angiogenic placental growth factor (PLGF), while the anti-angiogenic protein sFLT-1 hinders vascular endothelial growth factor (VEGF) signalling, crucial for maintaining vasorelaxation. Elevated levels of circulating sFLT-1 and reduced levels of pro-angiogenic factors create an anti-angiogenic state contributing to PE’s clinical manifestations [49]. A high plasma sFLT-1:PLGF ratio serves as a robust predictor of disease severity and adverse out-comes. Furthermore, placental oxidative stress, which is thought to be caused by mitochondrial dysfunction, is a feature of PE, affecting the secretion of cytokines, like IL-6 and IL-8 [43]. The transforming growth factor beta 1 (TGF-β1) is another cytokine that was shown to have an important role in PE’s development, since it inhibits trophoblast invasion of maternal arteries, possibly through an induction of tissue inhibitor of MMPs (matrix metalloproteinases) expression. This consequence, when severe, results in coexistent IUGR [50,51].

Current screening and diagnosis of PE rely on a comprehensive analysis of clinical, biochemical (Pregnancy-associated plasma Protein A (PAPP-A), Beta-Human Chorionic Gonadotropin (β-hCG), PLGF, sFLT-1), and biophysical (blood pressure, uterine artery Doppler measurement) parameters using a trimester-based risk algorithm developed by the Fetal Medicine Foundation, UK (FMF) [52]. While conventional biomarkers like PAPP-A and β-hCG, in combination with sFLT-1 and PLGF, are utilised for risk assessment, their sensitivity and disease selectivity can be enhanced. Recent research into circulating miRNAs in pregnant women shows promise as a minimally invasive method for predicting pregnancy-related complications, including PE, before clinical diagnosis. miRNA measurement also offers a non-invasive means to monitor fetal and maternal organ health. Enhancing the detection and utilization of miRNAs in pregnant women represents a significant area of research that could advance the prediction and management of pregnancy-related complications [53]. If these miRNAs can be integrated into the current clinical workflow, the ability to early diagnose, predict, monitor, and timely treat PE could be significantly improved.

### 4.5. Intrauterine Growth Restriction (IUGR)

Intrauterine growth restriction (IUGR, also known as fetal growth restriction) occurs when the foetus does not reach its intrauterine potential for growth and development as a result of placental dysfunction and/or maternal undernutrition [54,55]. This condition occurs in 5–10% of pregnancies, is responsible for 30% of stillbirths and is the most common cause of preterm births. Also, it is a marker of an adverse intrauterine environment and a strong predictor for the development of metabolic, cardiovascular, and renal diseases in adulthood [32,55]. It is well established that IUGR has a strong association with adverse long-term health consequences collectively described as the “metabolic syndrome”, including hypertension, coronary heart disease, visceral obesity (accumulation of excess fat especially in the abdominal cavity), impaired glucose tolerance and type 2 diabetes [32,55,56]. Furthermore, babies that are born small for gestational age (SGA, with a birth weight less than the 10th percentile for their gestational age) have often experienced IUGR [55].

Early IUGR (less than 32 weeks of gestation) is associated with substantial alterations in placental implantation and have higher rates of perinatal morbidity and mortality. On the other hand, late IUGR (more than 32 weeks of gestation) presents slight deficiencies in placentation and lower rates of perinatal morbidity and mortality [54]. In a study with pregnant rats, it was shown that inducing IUGR during the final trimester (days 14 to 20) is possible, and the effects can be attenuated by administering IL-10, indicating that IUGR relies, to some extent, on how placental cytokines, such as IL-10, interact and impact placental growth, development, or function [57,58].

### 4.6. Gestational Diabetes Mellitus (GDM)

Gestational diabetes mellitus (GDM) is one of the most common perinatal pathologies, with a prevalence of 5–20% depending on the population or diagnostic standards [59]. This disorder is defined as the onset of impaired glucose tolerance during pregnancy and occurs due to an impaired capacity of the maternal beta cells (pregnant woman’s specialised pancreatic cells) to adapt to the decreased insulin sensitivity that occurs during pregnancy [32]. This disorder leads to structural and functional changes in the placenta dependent on the modality and quality of the glycaemic control [34]. The increasing number of pregnant women developing GDM is closely related to the growing number of overweight and obese pregnant individuals, which often contributes to the delivery of large for gestational age (LGA) babies (babies born with higher percent body fat when compared to infants from pregnancies without medical complications) [34,55].

GDM has a strong relation with oxidative stress, which make mitochondrial dysfunction capable of influence it, increasing placental oxidative stress, and, in turn, affecting the secretion of IL-6 and IL-8 [43]. It has been hypothesised that differences in the placental microbiota could be associated with pregnancy complications, and one study showed that the placental microbiota from a women diagnosed with GDM differs from that of normoglycemic women, especially in terms of anti-inflammatory cytokines, like IL-10 [60]. Furthermore, GDM is associated with alterations in the concentration of CCL-4 and can lead to adverse short-term perinatal complications, such as PE and placental malfunction, as well as long-term metabolic disorder complications, such as type 2 diabetes mellitus in both offspring and mother [61].

## 5. Biomolecules

**Figure 1:**
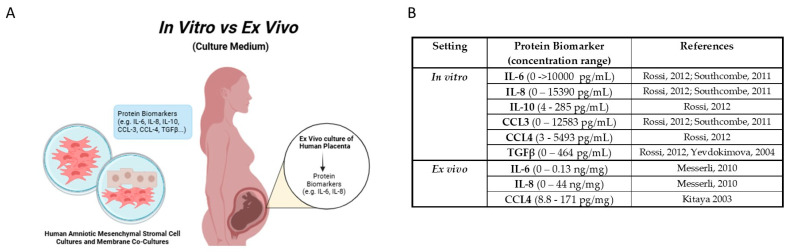
Biomolecules and In Vitro and Ex Vivo Experimental Analysis. (**A**). Schematic representation of in vitro and ex vivo biomolecules analysis during pregnancy. (**B**). Summary table highlighting the protein biomarkers and their concentration range detected through both in vitro and ex vivo methods [79,86,88,89,90].

**Figure 2:**
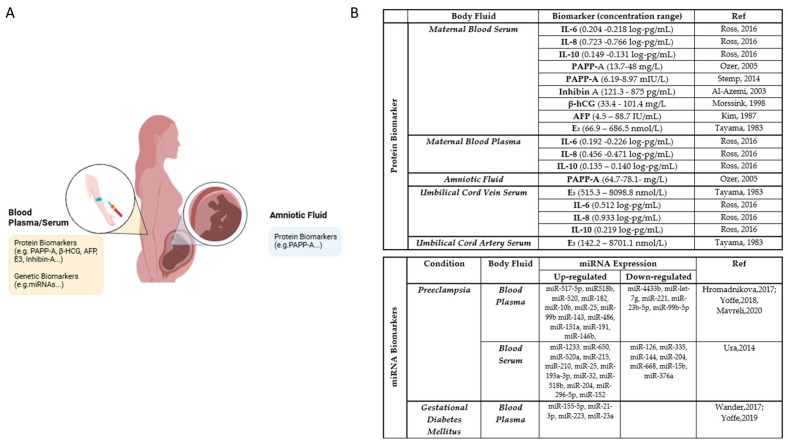
Biomolecules and Biological Fluid Analysis. (**A**). Schematic representation of body fluids and biomolecules analysed throughout pregnancy. (**B**). Summary table highlighting the protein and mRNA biomarkers, along with their concentration ranges, detected across different body fluids [76,91,92,93,94,95,96,97,98,99,100,101,102].

The previous discussion about molecular signatures in placenta-related diseases demonstrate that there are various molecules that have an important role in the regulation of placental function and can be used to detect placental complications, demonstrating they possible role as biomarkers for pregnancy complications. Most of these potential biomarkers (Figure 1 and Figure 2 and Table S1) are interleukins (IL-6 and IL-10), chemokines (IL-8, CCL-3 and CCL-4), and growth factors (TGF-β1, PLGF, and VEGF), which are bioactive molecules that play an important role in inducing physiological adaptations required for a successful pregnancy, however when inappropriately regulated they lead to disease [32].

Furthermore, proteins like sFlt-1, PAPP-A, β-hCG, and miRNA (Figure 2 and Table S2) are already being used as biomarkers for PE and need to be considered since combining the quantification of all these biomarkers could enhance the current screening and diagnosis of this disease.

Interleukins, such as IL-6 and IL-10, are small proteins released by immune cells that act via receptors to regulate the growth, maturation, and responsiveness of particular cell populations. These proteins are most well-known for the key role they play in regulation of the immune system, and therefore, cytokine pathways have been utilised as targets for successful therapeutic intervention via marketed products. Additionally, they are produced by a wide variety of immune and nonimmune cells and bind to cell surface receptors, triggering differential gene expression by the targeted cells, and forming complex interactive networks with potential autocrine, paracrine, and endocrine signalling [62]. IL-6 and IL-10, mediate the interactions between immune and inflammatory cells, and are able to promote cell growth, differentiation, and functional activation [63]. IL-6, a pro-inflammatory cytokine, is synthesized from T-lymphocytes, fibroblasts, endothelial cells, and monocytes [64,65]. A variety of infectious diseases can cause an increase in serum IL-6 levels, which is also associated with mortality [66]. IL-10, an anti-inflammatory cytokine, targets both innate and adaptive responses [67,68]. Also, it exerts immunosuppressive functions to reduce tissue damage caused by excess and uncontrolled inflammatory effector responses, especially during the resolution phase of infection and inflammation. This interleukin is produced by almost all subsets of leukocytes, including macrophages and B cells [68].

Chemokines (or chemotactic cytokines), such as IL-8, CCL-3 and CCL-4, are best known for stimulating cell migration and playing a central role in the development and homeostasis of the immune system. Additionally, they are involved in all protective or destructive immune and inflammatory responses [69]. IL-8 is a pro-inflammatory chemokine that, during an injury or infection, is involved in the recruitment of neutrophils from blood vessels to the affected tissue promoting angiogenesis, and stimulants, such as pro-inflammatory cytokines, cellular stress, or bacterial and viral products, trigger cells to produce it [70,71,72,73,74,75]. In a normal pregnancy, IL-8 concentration generally increases between the second and third trimesters [76]. However, during in vitro fertilization process, high levels of IL-8 correlate with incorrect embryo development and unsuccessful embryo implantation [77]. This finding goes well with the fact that the exposure of human amniotic mesenchymal cells to inflammatory stimuli upregulates the secretion of IL-8 [78,79]. Moreover, this chemokine also appears to be capable of suppressing spontaneous apoptosis in IL-8-expressing cells and has been implicated as a survival factor for endothelial cells [73,80]. CCL-3 and CCL-4 are also pro-inflammatory chemokines and highly related members of the C-C chemokine family and were shown capable of inducing chemotactic mobilization of monocyte-lineage cells and lymphocytes into inflammatory tissues [81,82]. CCL-3 guides and activates macrophages to secrete IL-6 [83]. Whereas CCL-4 demonstrate significant effects on cell apoptosis at a higher concentration (40 ng/mL), while at lower concentrations, generates no obvious effect [84].

Growth factors, specifically TGF-β superfamily, are known for their roles in cell proliferation, differentiation, apoptosis, and tissue remodelling. They are abundantly and dynamically expressed in the endometrium, and appear to have instrumental roles in modulating cellular events involved in placental hormone production, menstruation, proliferation, decidualisation, and the establishment of pregnancy [85]. Regarding TGF-β1, this protein mediates the growth and the cell cycle effects of high glucose in human umbilical vein endothelial cells [86]. Additionally, it is thought to participate in the induction of adult mesenchymal stem cells (MSCs)-mediated immunosuppression [87].

## 6. Future Trends and Innovations in Maternal-Fetal Health

The growing concern over mortality and morbidity rates related to placenta dysfunction and related diseases has led to an increased demand for validation and discovery of predictive molecular biomarkers. Measuring clinically relevant biomarkers in readily available body fluids is an unmet need for early and improved diagnosis and monitoring of pregnant women to accelerate decision-making with the placental dysfunction risk development. Reliable point-of-care (POC) tests for biomarkers is essential for rapid and informed clinical decisions, as delaying diagnosis puts both the mother and foetus at risk. However, dependable POC testing for key biomarkers that can accelerate early diagnosis, aid in pregnancy risk assessment, and monitoring before symptoms manifest remains an unmet need. Addressing this unmet need in the near future can significantly improve maternal and fetal outcomes, reducing the burden of these conditions on healthcare systems and society as a whole.

Current in vitro diagnostic technologies mainly include molecular biology diagnosis, immunoassay, and physiological signal monitoring. Among these technologies, immunoassays have been routinely used to detect various biomarkers, such as small molecules, proteins, and nucleic acids [103]. ELISA has become the most popular immunoassay because of its good specificity, good sensitivity, and high throughput [104]. The readout of the results from conventional ELISA relies on the naked eye or a plate reader, which allows a faster quantitative analysis of the absorbance or fluorescence results. However, the huge size of plate reader normally limits the use of ELISA for POC applications [105,106]. Also, these conventional immunoassay such as ELISA require complex operation processes and expensive instruments, making the use of this in a POC system even more difficult [107]. To enable a better and more efficient detection of these biomarkers for pregnancy complications, it would be better to have a faster and easier way to do it, creating a diagnostic technique that could be use in a POC context with ease.

While additional studies are needed to validate the potential of the described biomarkers, alongside clinical symptoms, for the diagnosis and monitoring of pregnancy complications, this review underscores the consistent alterations of these biomolecules across various pregnancy-related pathologies, highlighting their relevance.

## 7. Conclusions

In conclusion, the biomarkers discussed above show great promise as biomarkers for pregnancy complications. Incorporating these biomarkers into a POC system, which can be utilized by the doctor during medical appointments, would offer pregnant women a fast and easy way to know if their pregnancy and, with it, their baby are at risk or not.

Thus, decreasing the number of preterm births and giving a better quality of life for the mother and the fetus.

## Data Availability

Not applicable.

## Supplementary Materials

The following supporting information can be downloaded at: https://www.mdpi.com/article/10.3390/biom15030312/s1, Table S1: Concentrations of biomarkers during different pregnancy and placenta cell-line conditions [107]; Table S2: First trimester extracellular miRNAs during different pregnancy conditions [97,98,99,100,101,102].
