# Supplementary material for: Molecular Biomarkers for Timely and Personalized Prediction of Maternal-Fetal Health Risk
Source: Biomolecules. 2025 Feb 20;15(3):312. doi: 10.3390/biom15030312 (PMC11940122; doi:10.3390/biom15030312)
Supplement: Supplementary file 1 [file biomolecules-15-00312-s001.zip › biomolecules-3354721-supplementary.pdf]

Table 1. Concentrations of biomarkers during different pregnancy and placenta cell-line conditions.

Condition	Study design	Detection Method	Concentration biomarker	Ref
Ultraculture medium	n ≥ 3	Multiple cytometric beads array system	IL-6 - 0 pg/mL<br>IL-8 – 0 pg/mL<br>IL-10 – 4 pg/mL<br>CCL3 – 0 pg/mL<br>CCL4 – 3 pg/mL<br>TGFβ – 0 pg/mL	[79]
Culture medium from human amniotic mesenchymal tissue cells (CM-hAMTC)			IL-6 - >10000pg/mL<br>IL-8 – 2591 pg/mL<br>IL-10 – 285 pg/mL<br>CCL3 – 12583 pg/mL<br>CCL4 – 3838 pg/mL<br>TGFβ – 464 pg/mL	
Culture medium from human amniotic membrane (CM-hAM)			IL-6 ->10000 pg/mL<br>IL-8 – 11527 pg/mL<br>IL-10 – 9 pg/mL<br>CCL3 – 7368 pg/mL<br>CCL4 – 5493pg/mL<br>TGFβ – 209pg/mL	
Human Placental tissue - Incubation was performed in 20%O2/5% CO2 (eS_{20})	n = 5	ELISA	IL-6 - 0.13 ng/mg<br>IL-8 – 44.0 ng/mg	[88]
Human Placental tissue - Placental dual perfusion (pS)	n = 3		IL-6 - 0.0 ng/mg<br>IL-8 – 0 ng/mg	
Human Placental tissue - Incubation in 3 % O_2/5 % CO_2 (eS_3)	n = 3		IL-6 - 0.07 ng/mg<br>IL-8 – 0.7 ng/mg	
Human Placental tissue - Mechanical dissection (mS)	n = 4		IL-6 - 0 ng/mg<br>IL-8 – 0.07 ng/mg	
Blood from Stress, Pregnancy and Health study (SPAH) (2nd trimester)	n = 73-100	Electrochemo luminescent immune assay (ECLIA)	IL-6 - 0.204 log-pg/mL<br>IL-8 – 0.723 log-pg/mL<br>IL-10 -0.149 log-pg/mL	[76]
Blood from SPAH (3rd trimester)	n = 70-93		IL-6 - 0.218 log-pg/mL<br>IL-8 – 0.766 log-pg/mL<br>IL-10–0.131 log-pg/mL	
Blood from SPAH (Cord blood)	n = 87		IL-6 - 0.512 log-pg/mL<br>IL-8 – 0.933 log-pg/mL<br>IL-10–0.219 log-pg/mL	
Blood from Measurement of Maternal Stress study (MOMS) (2nd trimester)	n = 519-539		IL-6 - 0.192 log-pg/mL<br>IL-8 – 0.456 log-pg/mL<br>IL-10–0.135 log-pg/mL	
Blood from MOMS (3rd trimester)	n = 522-538		IL- 6 - 0.226 log-pg/mL<br>IL-8 – 0.471 log-pg/mL<br>IL-10–0.140 log-pg/mL	
PBMC from non-pregant women + Syncytiotrophoblast microvesicles (STBM) obtained by perfusion from the maternal side of a placenta lobe (pSTBM)	n = 10	ELISA	IL-6 - 8487 pg/mL<br>IL-8 – 15390 pg/mL<br>CCL3 – 812.8 pg/mL	[89]
PBMC from pregnant women (3rd trimester) + pSTBM			IL-6 - 5980 pg/mL<br>IL-8 – 1040 pg/mL<br>CCL3 – 9.609 pg/mL	

PBMC from pregnant women (3rd trimester) + STBM derived from mechanical dissection of the placenta (mSTBM)			IL-6 – 0 pg/mL<br>IL-8 – 10160 pg/mL<br>CCL3 – 497.5 pg/mL	
Endometrial stromal cells (ESC) Early proliferative phase (EP) (4-7 days)	n = 7	ELISA	CCL4 - 8.8 ± 12.5 pg/mg protein	[90]
ESC Mid-proliferative phase (8-11 days) (MP)	n = 9		CCL4 - 31.1 ± 16.3 pg/mg protein	
ESC: Late proliferative phase (12-14 days) (LP)	n = 6		CCL4 - 55.6 ± 38.9 pg/mg protein	
ESC Early secretory phase (15-18 days) (ES)	n = 9		CCL4 - 140.9 ± 51.6 pg/mg protein	
ESC Mid-secretory phase (19-23 days) (MS)	n = 10		CCL4 - 162.1 ± 61.3 pg/mg protein	
ESC Late secretory days (24 days onward) (LS)	n = 11		CCL4 - 171.0 ± 61.5 pg/mg protein	
Human endothelial cell line (ECV304) Total with 5.6 mM glucose	n = 12	MvlLu cell Growth inhibition assay	TGFβ - 7.71 pg/mL	[86]
Total ECV304 with 30 mM glucose	n = 12		TGFβ -10.64 pg/mL	
Total ECV304 with 5.6 mM glucose + 25.4 mM mannitol	n = 10		TGFβ -8.10 pg/mL	
Active ECV304 with 5.6 mM glucose	n = 12		TGFβ -1.19 pg/mL	
Active ECV304 with 30 mM glucose	n = 12		TGFβ -1.91 pg/mL	
Active ECV304 with 5.6 mM glucose + 25.4 mM mannitol	n = 12		TGFβ -1.16 pg/mL	
Amniotic fluid (Term delivery (TD))	n = 127	ELISA	PAPP-A - 78.1 mg/L	[91]
Maternal serum (TD)	n = 127		PAPP-A - 48.0 mg/L	
Amniotic fluid (Preterm delivery (PD))	n = 14		PAPP-A - 64.7 mg/L	
Maternal serum (PD)	n = 14		PAPP-A - 13.7 mg/L	
Blood serum (Natural Early follicular (EF), 10 days	n = 10	Roche cobas e411 automated analyser (Roche Diagnostics, Germany)	PAPP-A - 8.97 mIU/L	[92]
Blood serum (Natural Mid-follicular (MF), 12 days	n = 10		PAPP-A - 8.91 mIU/L	
Blood serum (Natural Late follicular (LF), 13 days	n = 10		PAPP-A - 8.59 mIU/L	
Blood serum (Natural Mid-luteal (ML), 15 days	n = 10		PAPP-A - 8.40 mIU/L	
Blood serum (Stimulated EF, 12 days)	n = 11		PAPP-A - 6.24 mIU/L	
Blood serum (Stimulated MF, 13 days)	n = 11		PAPP-A - 6.73 mIU/L	
Blood serum (Stimulated LF, 14 days)	n = 11		PAPP-A - 6.57 mIU/L	
Blood serum (Stimulated ML, 15 days)	n = 11		PAPP-A - 6.19 mIU/L	
Maternal serum (8 weeks Gestational age (GA))	n = 6	ELISA	β-hCG - 101.4 mg/L	[93]
Maternal serum (9 weeks GA)	n = 103		β-hCG - 49.6 mg/L	
Maternal serum (10 weeks GA)	n = 401		β-hCG - 39.9 mg/L	
Maternal serum (11 weeks GA)	n = 122		β-hCG - 34.6 mg/L	
Maternal serum (12 weeks GA)	n = 8		β-hCG - 33.4 mg/L	
Maternal peripheral vein serum (MPVS) during labour (Baby weight appropriate, AFD) (Unconjugated estriol, uE_3)	n = 8	Oestriol (unconjugated) RIA kit	E_3 - 83.6 nmol/L	[94]
Infant cord vein serum (ICVS) at birth (AFD) (uE_3)	n = 15		E_3 - 515.3 nmol/L	
Infant cord artery serum (ICAS) at birth (AFD) (uE_3)	n = 15		E_3 - 184.1 nmol/L	
MPVS during labour (babies weight, at or lower than the tenth percentile of weight/gestational age, LFD) (uE_3) with IUGR	n = 3		E_3 - 66.9 nmol/L	
ICVS at birth (LFD) (uE_3) with (Intrauterine growth restriction, IUGR)	n = 6		E_3 - 368.2 nmol/L	

ICAS at birth (LFD) (uE_3) with IUGR	n = 6		E_3 - 142.2 nmol/L	
MPVS during labour (AFD) (Estriol, E_3)	n = 8	Oestriol RIA kit	E_3 - 655.3-2211.1 nmol/L	[94]
ICVS at birth (AFD) (E_3)	n = 15		E_3 - 8098.8 nmol/L	
ICAS at birth (AFD) (E_3)	n = 15		E_3 - 8701.1 nmol/L	
MPVS during labour (LFD) (E_3) with IUGR	n = 3		E_3 - 686.5 nmol/L	
ICVS at birth (LFD) (E_3) with IUGR	n = 6		E_3 - 6113.7 nmol/L	
ICAS at birth (LFD) (E_3) with IUGR	n = 6		E_3 - 5366.2 nmol/L	
Maternal serum (10 weeks GA)	n = 4	ELISA	AFP - 4.5 IU/mL	[95]
Maternal serum (12 weeks GA)	n = 11		AFP -10.0 IU/mL	
Maternal serum (13 weeks GA)	n = 10		AFP -7.0 IU/mL	
Maternal serum (14 weeks GA)	n = 14		AFP -21.5 IU/mL	
Maternal serum (15 weeks GA)	n = 16		AFP -23.5 IU/mL	
Maternal serum (16 weeks GA)	n = 17		AFP -31.0 IU/mL	
Maternal serum (17 weeks GA)	n = 12		AFP - 37.5 IU/mL	
Maternal serum (18 weeks GA)	n = 17		AFP - 32.5 IU/mL	
Maternal serum (19 weeks GA)	n = 7		AFP -60.0 IU/mL	
Maternal serum (20 weeks GA)	n = 8		AFP -67.5 IU/mL	
Maternal serum (21 weeks GA)	n = 8		AFP - 57.5 IU/mL	
Maternal serum (22 weeks GA)	n = 13		AFP - 88.7 IU/mL	
Blood serum (control group - Patients whose pregnancy continued beyond 20 weeks) (6 weeks GA)	n = 24	ELISA	Inhibin A - 429.4 pg/mL	[96]
Blood serum (control group) (8 weeks GA)	n = 24		Inhibin A - 725.0 pg/mL	
Blood serum (control group) (10 weeks GA)	n = 24		Inhibin A - 875.0 pg/mL	
Blood serum (control group) (12 weeks GA)	n = 24		Inhibin A - 727.5 pg/mL	
Blood serum (aborted group - Patients who aborted before 20 weeks) (6 weeks GA)	n = 12		Inhibin A - 234.5 pg/mL	
Blood serum (aborted group) (8 weeks GA)	n = 12		Inhibin A - 162.5 pg/mL	
Blood serum (aborted group) (10 weeks GA)	n = 12		Inhibin A - 121.3 pg/mL	
Blood serum (aborted group) (12 weeks GA)	n = 12		Inhibin A - 135.0 pg/mL	

Table 2. First trimester extracellular miRNAs during different pregnancy conditions

Condition	Study design	Detection Method	miRNAs differentially expressed	Ref
Preeclampsia (PE)	n = 21	Quantitative Real-Time Reverse Transcription PCR (qRT-PCR)	miR-517-5p, miR518b, miR-520h	[97]
PE	n = 24	Microarray analysis + qRT-PCR	miR-1233, miR-650, miR-520a, miR-215, miR-210, miR-25, miR-193a-3p, miR-32, miR-518b, miR-204, miR-296-5p, miR-152, miR-126, miR-335, miR-144, miR-204, miR-668, miR-15b, miR-376a	[98]
Early-PE	n = 35	RNA Sequencing	miR-182, miR-10b, miR-25, miR-99b, miR-4433b, miR-143, miR-let-7g, miR-486, miR-151a, miR-191, miR-146b, miR-221	[99]
Late-PE	n = 5	miRNA Deep Sequencing + qRT-PCR	miR-23b-5p, miR-99b-5p	[100]
Gestational Diabetes Mellitus (GDM)	n = 36	qRT-PCR	miR-155-5p, miR-21-3p	[101]
GDM	n = 23	Multiplexed NanoString nCounter miRNA expression assay + qRT-PCR	miR-223, miR-23a	[102]
